# The receptor for urokinase-plasminogen activator (uPAR) controls plasticity of cancer cell movement in mesenchymal and amoeboid migration style

**DOI:** 10.18632/oncotarget.1754

**Published:** 2014-03-10

**Authors:** Francesca Margheri, Cristina Luciani, Maria Letizia Taddei, Elisa Giannoni, Anna Laurenzana, Alessio Biagioni, Anastasia Chillà, Paola Chiarugi, Gabriella Fibbi, Mario Del Rosso1

**Affiliations:** ^1^ Department of Experimental and Clinical Biomedical Sciences, University of FlorenceIstituto Toscano Tumori

**Keywords:** uPAR, mesenchymal movement, amoeboid movement, prostate carcinoma, melanoma,Rho-GTPases

## Abstract

The receptor for the urokinase plasminogen activator (uPAR) is up-regulated in malignant tumors. Historically the function of uPAR in cancer cell invasion is strictly related to its property to promote uPA-dependent proteolysis of extracellular matrix and to open a path to malignant cells. These features are typical of mesenchymal motility. Here we show that the full-length form of uPAR is required when prostate and melanoma cancer cells convert their migration style from the “path generating” mesenchymal to the “path finding” amoeboid one, thus conferring a plasticity to tumor cell invasiveness across three-dimensional matrices. Indeed, in response to a protease inhibitors-rich milieu, prostate and melanoma cells activated an amoeboid invasion program connoted by retraction of cell protrusions, RhoA-mediated rounding of the cell body, formation of a cortical ring of actin and a reduction of Rac-1 activation. While the mesenchymal movement was reduced upon silencing of uPAR expression, the amoeboid one was almost completely abolished, in parallel with a deregulation of small Rho-GTPases activity. In melanoma and prostate cancer cells we have shown uPAR colocalization with β1/β3 integrins and actin cytoskeleton, as well integrins-actin co-localization under both mesenchymal and amoeboid conditions. Such co-localizations were lost upon treatment of cells with a peptide that inhibits uPAR-integrin interactions. Similarly to uPAR silencing, the peptide reduced mesenchymal invasion and almost abolished the amoeboid one. These results indicate that full-length uPAR bridges the mesenchymal and amoeboid style of movement by an inward-oriented activity based on its property to promote integrin-actin interactions and the following cytoskeleton assembly.

## INTRODUCTION

Cancer cells move within tissues collectively or as single cells. In collective invasion inter-cellular cohesion in form of strands, sheets, amorphous masses or more or less regularly shaped tubes is required during the invasion process [1]. While collective movement has been associated with tumor cell entry into the open lymphatic vessels, the movement of individual cells is required for cancer cells to sneak across basement membranes, to enter the bloodstream and disseminate to distant organs [2]. The invasion strategy of single tumor cells develops according to at least two distinct modes of migration: the so-called elongated-mesenchymal mode and rounded-amoeboid mode. The mesenchymal migration requires the activity of extracellular matrix (ECM) degrading proteases, while amoeboid motility is an opportunistic movement, which allows cells to glide through, rather than degrade, ECM barriers, using movements based on adaptations of the cell body. The mesenchymal movement depends on Rac-driven actin cytoskeleton contractility, while the amoeboid one relies on Rho-ROCK-dependent regulation of cortical actin to generate cortical tension, stiffness and the maintenance of roundish cell morphology [3]. Single “default” migration styles are preferentially employed by a particular cell, such as the amoeboid for leukocytes, the mesenchymal for stromal cells, the collective for epithelial cell sheets [2]. The most widespread mean to promote an amoeboid movement pattern in cancer cells cultured *in vitro* is to create an artificial environment where cells must cope with a mixture of protease inhibitors [4]. Little is known about physiological inducers of amoeboid motility. In many experimental examples, mesenchymal-to-amoeboid transition (MAT) depends on pathways that weaken Rac and/or strengthen Rho/ROCK signalling [1-5]. Epigenetic expression of regulating factors seems to be important. In particular, pathways that activate Rho lead to MAT, including inhibition of negative Rho regulators [6] or the activation of Ephrin2A receptor tyrosine kinase signalling [7]. Among environmental conditions, an interesting paper has highlighted the role of matrix-bound plasminogen inhibitor type-1 (PAI-1) in supporting amoeboid movement and cell blebbing of human colorectal cancer cells via RhoA/ROCK1 signaling [8].

Experimental evidences accumulated over the last 25 years connote the receptor for the urokinase-type plasminogen activator (uPAR) as the prototype receptor regulating the mesenchymal style of cell movement by triggering pericellular proteolysis of invasive cells. Plasmin generated following the direct activation of plasminogen by uPAR-bound urokinase plasminogen activator (uPA) opens a path to invasive cells by both direct and pro-matrix-metallo-proteinases (MMPs)-activation-dependent degradation of ECM. Besides plasmin-generation-dependent activities uPAR also shows proteolysis-independent functions. Such properties entail uPAR interaction with vitronectin (VN) and integrins, able to provide a foothold to moving cells [9]. In particular, uPAR interaction with integrins has been shown to increase integrin affinity for ECM ligands [10]. Besides outward-oriented activities, uPAR is also involved in the regulation of the actin cytoskeleton and cell motility [11]. Due to its glycosyl-phosphatidyl-inositol (GPI)-anchor attachment [12], uPAR is devoid of a cytoplasmic domain, a feature that renders uPAR incapable of signalling. This characteristic requires membrane partners enabling uPAR to deliver signals that propagate to the cell contractile apparatus. Most consistently uPAR has been found associated with integrins [11,13,14]. Besides interaction with ECM ligands, integrins provide a molecular link that connects microenvironment to the cytoskeleton. Together with a long series of adaptor proteins integrins define molecular mechanical pathways in cells, which subsequently determine actin dynamics and cell movement [15]. Thus, a major pathway that controls in-ward integrin activity may define and distinguish cancer cell invasion strategies. On these considerations, uPAR appears a good candidate molecule capable of modulating integrin function and to sustain the style of movement of a cell. Here we show that uPAR bridges the mesenchymal and amoeboid style of movement in a series of prostate carcinoma and melanoma cell lines, by its property to warrant the integrin-mediated connection between actin cytoskeleton and the cell membrane.

## RESULTS

### Function of uPAR in mesenchymal invasion of tumor cells

**Figure 1:**
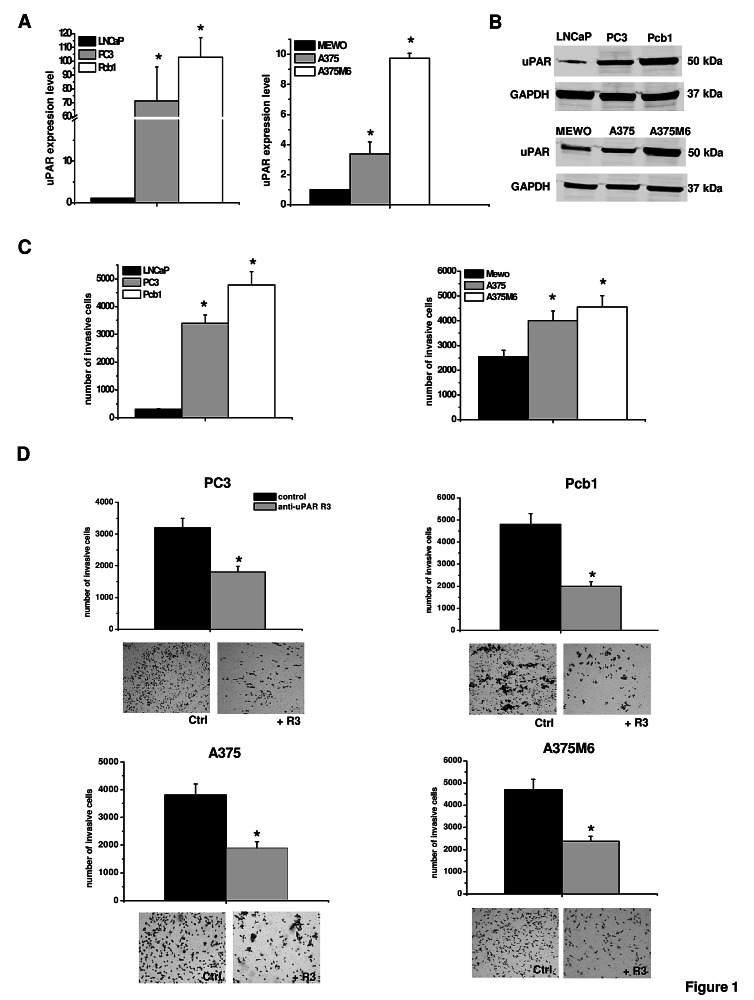
uPAR expression and function in mesenchymal invasion of prostate cancer and melanoma cells Panel A: Quantitative Real-Time PCR of uPAR in melanoma and prostate cancer cell lines. Results are the mean of three different experiments ± SD. *: p<0.05 with respect to LNCaP and MEWO cells, respectively. Panel B: Western Blotting analysis of uPAR in prostate cancer and melanoma cell lines. Numbers on the right refer to molecular weights expressed in kDa. GAPDH used as a reference loading control. Panel C: invasion of porous filters coated with a 3D Matrigel layer, showing the differential total number of invading cells/filter for each one of the used cell lines. Panel D: activity of the R3 uPAR-blocking antibody on Matrigel invasion. The pictures show the typical appearance of filters in the absence and in the presence of the R3 antibody for each cell line. Results are the mean of three different experiments ± SD. * : p<0.05 with respect to LNCaP and MEWO cells (panel B), or with respect to control (panel C), respectively.

uPAR is expressed by cells that move in a mesenchymal fashion. uPAR-bound uPA promotes plasminogen activation to plasmin and subsequent pro-MMPs activation-dependent ECM degradation. To verify the role of uPAR in the mesenchymal movement we measured uPAR by a quantitative Real-Time PCR and Western Blotting analysis showing that uPAR expression was proportional to the number of invasive cells in the Boyden chamber-Matrigel invasion assay (figures 1A, 1B and 1C). Blocking uPAR mesenchymal function with an antibody (R3) against uPAR domain-1, thereby masking the uPA binding site, resulted into a decrease of invasion which was similar in all the examined cancer cell lines (figure 1D). The use of the blocking antibody blunted the uPAR-dependent proteolytic cascade without using protease inhibitors. In the absence of protease inhibitors the pre-specified movement style of single migrating cancer cells is the mesenchymal one [2].

### Induction of the amoeboid phenotype

**Figure 2:**
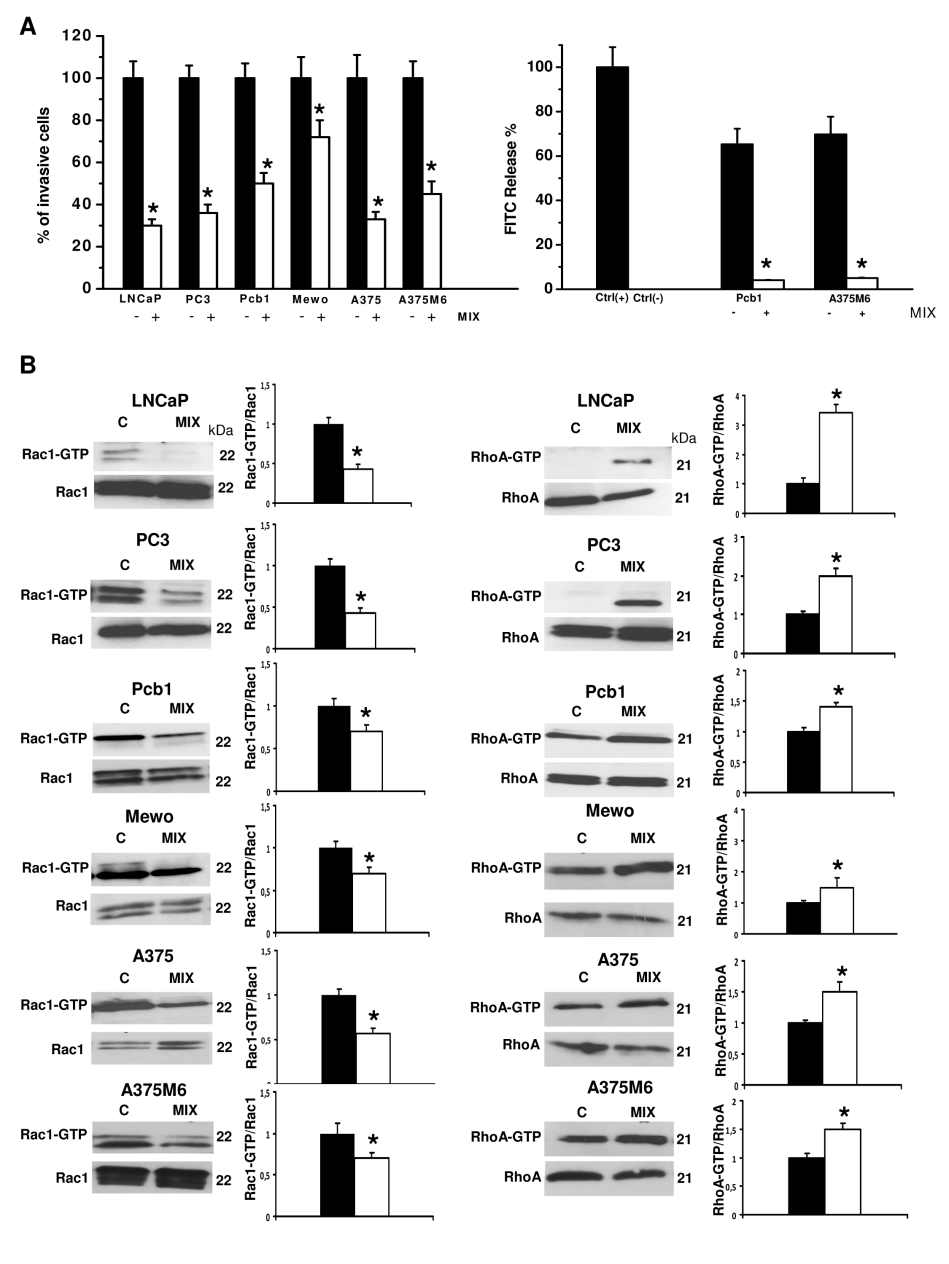
Induction of the amoeboid movement in prostate cancer and melanoma cells Panel A: the histogram on the left shows invasion of porous filters coated with a 3D Matrigel layer, expressed as % cells moving under amoeboid conditions for each one of the cell lines. Refer to figure 1 for the absolute number of invading cells under mesenchymal conditions. Mix: presence of the protease inhibitors cocktail. Results are the mean of five different experiments performed in triplicate on each cell line ± SD. * : p<0.05. The histogram on the right shows the collagenolytic activity of Pcb1 and A375M6 cells under mesenchymal and amoeboid conditions, expressed as % collagen degradation with respect to the positive control obtained by addition of exogenous collagenase. Ctrl-: collagenolytic activity in the absence of cells and of added exogenous collagen; Ctrl+: collagenolytic activity in the absence of cells but in the presence of exogenous collagenase. Results are the mean of two different experiments performed in triplicate on each cell line ± SD. * : p<0.05. Panel B: Western blotting of total and GTP-loaded forms of small Rho-GTPases RhoA and Rac1 under mesenchymal and amoeboid conditions for each prostate cancer and melanoma cell line. Three experiments were performed for each cell line in each condition. Histograms report band densitometry; * indicates statistical significance at p<0.05. Numbers on the right refer to molecular weights expressed in kDa. Rac1-GTP and RhoA-GTP, GTP-loaded forms of small Rho GTP-ases; Rac1 and RhoA, total un-loaded forms of small Rho GTP-ases, used as a reference loading control.

**Figure 3:**
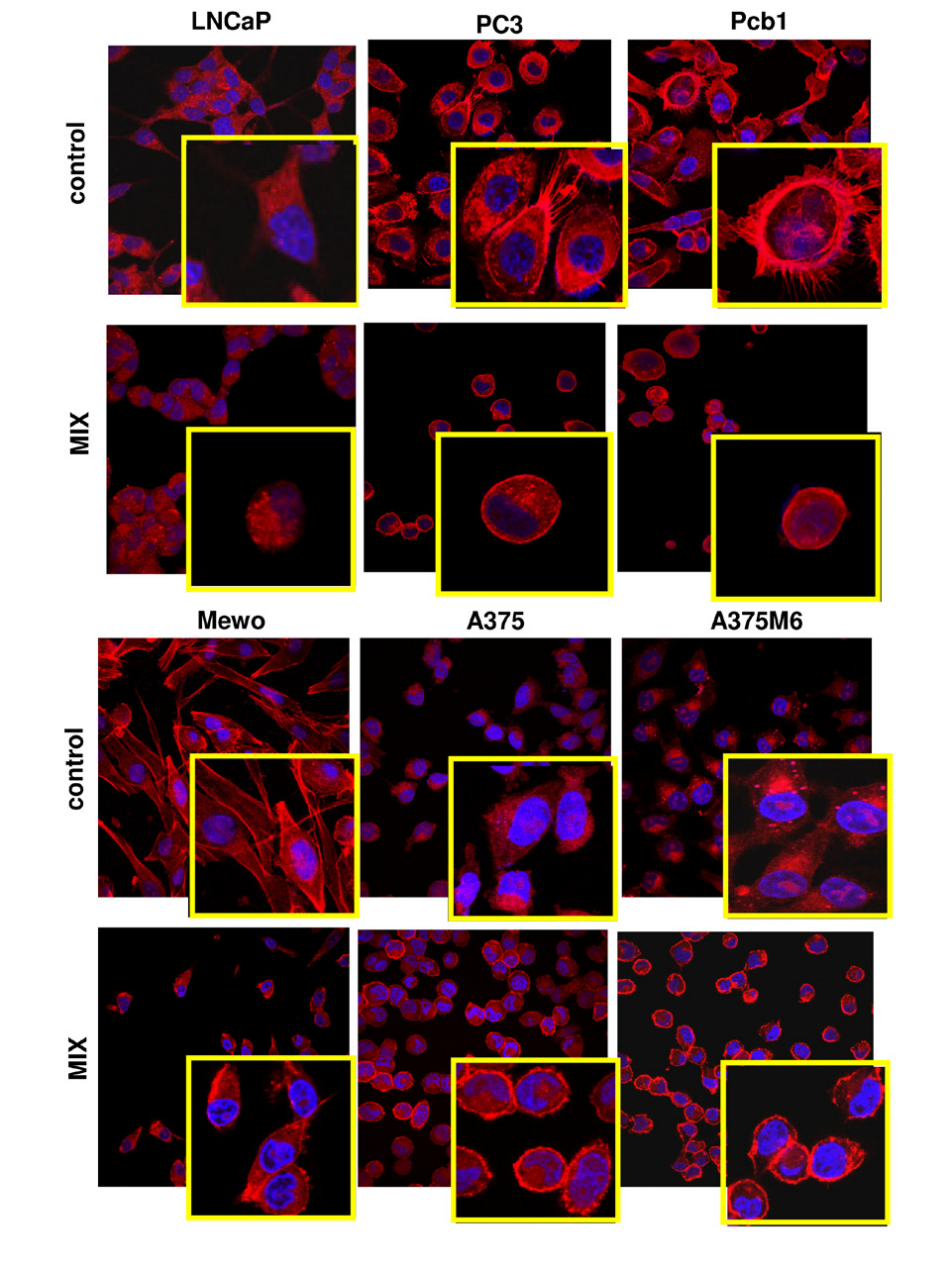
Morphological features of the mesenchymal (elongated) to amoeboid (roundish) transition (MAT) of prostate cancer and melanoma cells Each picture shows the general pattern and related magnification of a small field for each cell line. Red: phalloidin staining of the actin cytoskeleton. Blue: nuclear staining with DAPI. Magnification 40 X for reference pictures and 100 X for enlarged insets. Results shown are representative of two different preparations of each cell line under mesenchymal and amoeboid conditions. Round cells, perinuclear cortical actin localization and the loss of actin-rich filopodia are evident in amoeboid conditions.

Matrigel invasion, Rac1/RhoA activation and cell morphology*.* To evaluate whether exposure to a protease inhibitors cocktail induced a protease-independent invasion, we subjected cancer cell lines to a Boyden chamber migration assay through a thick Matrigel coating, in the presence of the inhibitor cocktail added to the cell suspension and to Matrigel solution before polymerization. The percent amoeboid response of each cell line with respect to the mesenchymal control was not comparable in terms of invasive cells, ranging from 30% to 72% depending on the cancer cell line, indicating that plasticity of cell migration upon variation of microenvironment conditions is greatly variable among cancer cells (figure 2A). The experimental conditions here referred to as “amoeboid” were characterized by a residual proteolytic activity that did not account for the size of the invasive cell population (figure 2A). To characterize the motility shift we also analyzed the activation of two recognized regulators of the cytoskeleton, the small GPTases RhoA and Rac1, and cell morphology, as related to actin organization. Mesenchymal motility has been recognized to be causally linked to activation of Rac, which drives motility by promoting lamellipodia and filopodia, and inhibition of Rho GTPases, whereas an opposite phenotype has been associated with amoeboid motility [16,17]. Western blotting data for the activated forms of RhoA and Rac1, compared to total RhoA and Rac1, provided univocal results for all the examined cell lines, showing a decrease of activated Rac1 and an increase of activated RhoA upon shifting of cells to the amoeboid movement (figure 2B). The overall morphology of the cell and its dependency on actin cytoskeleton assembly is characteristic of the movement style. Mesenchymal motility is connoted by an elongated, fibroblast-like, cell morphology with established cell polarity, dependent on the small GTPase Rac which, in turn, organizes actin polymerization to form filopodia and lamellipodia [18], giving origin to actin-rich protrusions. These features were exhibited by cancer cells under control conditions. Under the spur of protease inhibition all the cell lines acquired a round morphology and a sub-membranous cortical actin localization, features connoting the amoeboid movement (figure 3). The WST-1 assay showed that viable cells were more that 95% under both control and protease inhibitors treatment. Moreover, once re-incubated with an inhibitor cocktail-free medium, treated cells completely recovered their functional and morphological mesenchymal features (not shown).

### Requirement of uPAR in amoeboid migration

**Figure 4:**
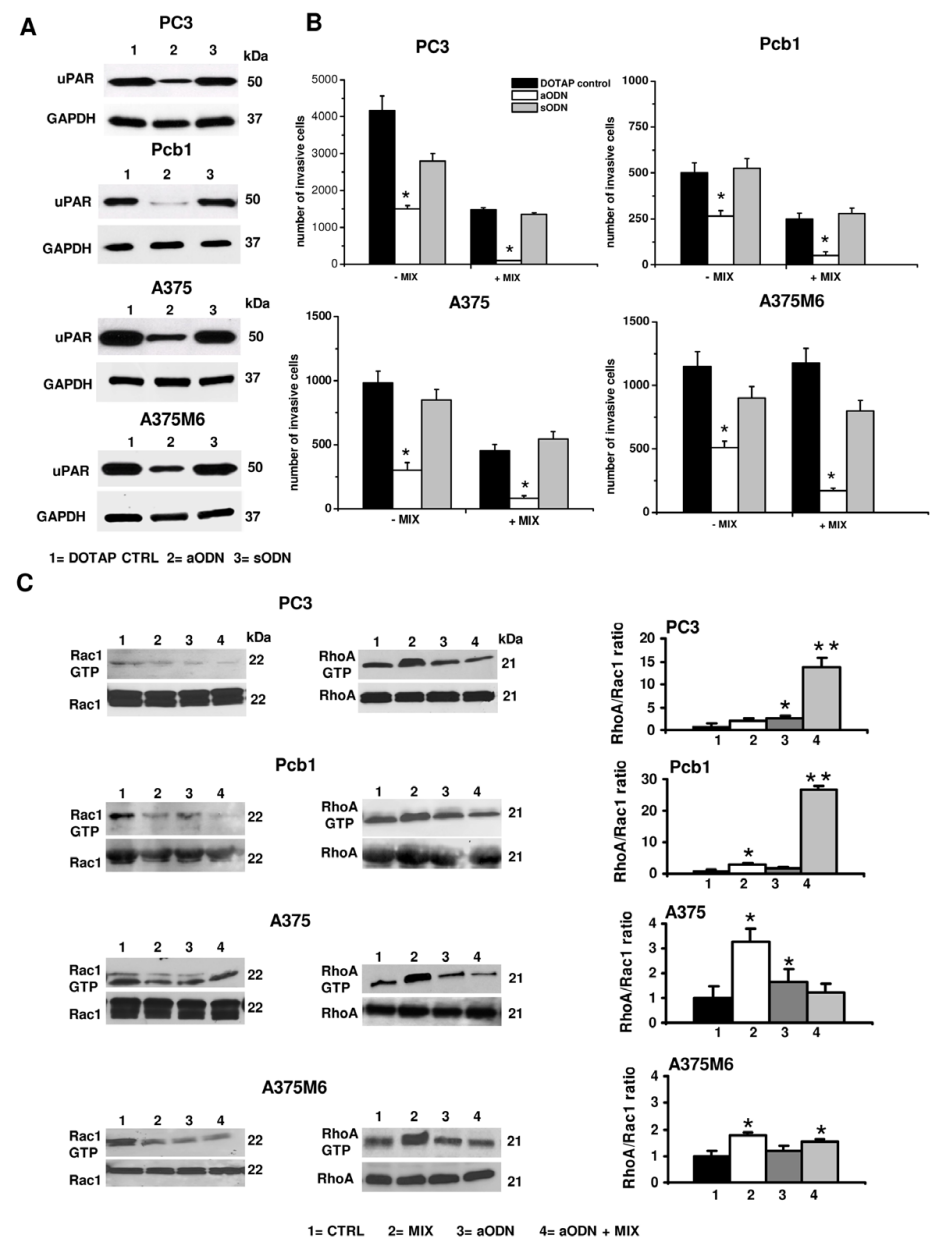
Effects of uPAR silencing with uPAR-aODN on invasion and small Rho-GTPases activation in mesenchymal and amoeboid conditions of prostate and melanoma cancer cells Panel A: Western Blotting analysis of uPAR for each prostate cancer and melanoma cell line after uPAR-aODN treatment. DOTAP: treatment of cells with the cationic liposome alone; aODN: treatment of cells with DOTAP liposomes-delivered uPAR-antisense oligodeoxynucleotide; sODN: treatment of cells with DOTAP liposomes-delivered scramble oligodeoxynucleotide. Numbers on the right of each Western blotting refer to molecular weights expressed in kDa. GAPDH used as a reference loding control. Panel B: Matrigel invasion under mesenchymal (-MIX) and amoeboid (+MIX) conditions. Results are the mean of three experiments performed in triplicate on each cell line ± SD. * : p<0.05 with respect to controls. Panel C: Western blotting of total and GTP-loaded forms of small Rho-GTPases RhoA and Rac1 under mesenchymal and amoeboid conditions for each prostate cancer and melanoma cell line, untreated and treated with aODN/sODN, respectively. Histograms report RhoA/Rac1 ratio obtained by band densitometry quantification. *: statistical significance at p<0.05 with respect to control; **: statistical significance at p<0.01 with respect to controls. Numbers on the right of each Western blotting refer to molecular weights expressed in kDa. For other symbols, refer to the legend of figure 1.

Genetic uPAR knockdown has demonstrated robust antitumor activity both *in vitro* and in pre-clinical studies [19]. To induce the loss of uPAR function we used the uPAR-aODN previously used in other studies of our laboratory [20-22]. uPAR-aODN produced an evident reduction of uPAR expression inducing a strong decrease of mesenchymal and amoeboid movements (figure 4A and 4B). Both in control and in the presence of protease inhibitors all the cell lines showed a reduced active Rac1 paralleled by an increased RhoA activation, while uPAR-aODN treatment resulted into reduced Rac1 activation either in the absence or in the presence of the inhibitor cocktail. The RhoA/Rac1 ratio in the presence of aODN and aODN + inhibitor cocktail conformed to the classical amoeboid pattern indicating a prevalence of RhoA activity in prostate cancer cell lines, while it showed smaller variations with respect to controls in melanoma cell lines (figure 4C). Therefore, under amoeboid conditions and in the presence of uPAR knockdown, cancer cells still activate ameoboid-related transductions but are unable to invade 3D matrices, indicating that the loss of uPAR is detrimental for both movement styles. The residual movement observed in the absence of protease inhibitors in control cells treated with uPAR-aODN must be ascribed to proteases of different families.

### The full-length D1D2D3 form of uPAR is required to induce amoeboid migration.

**Figure 5:**
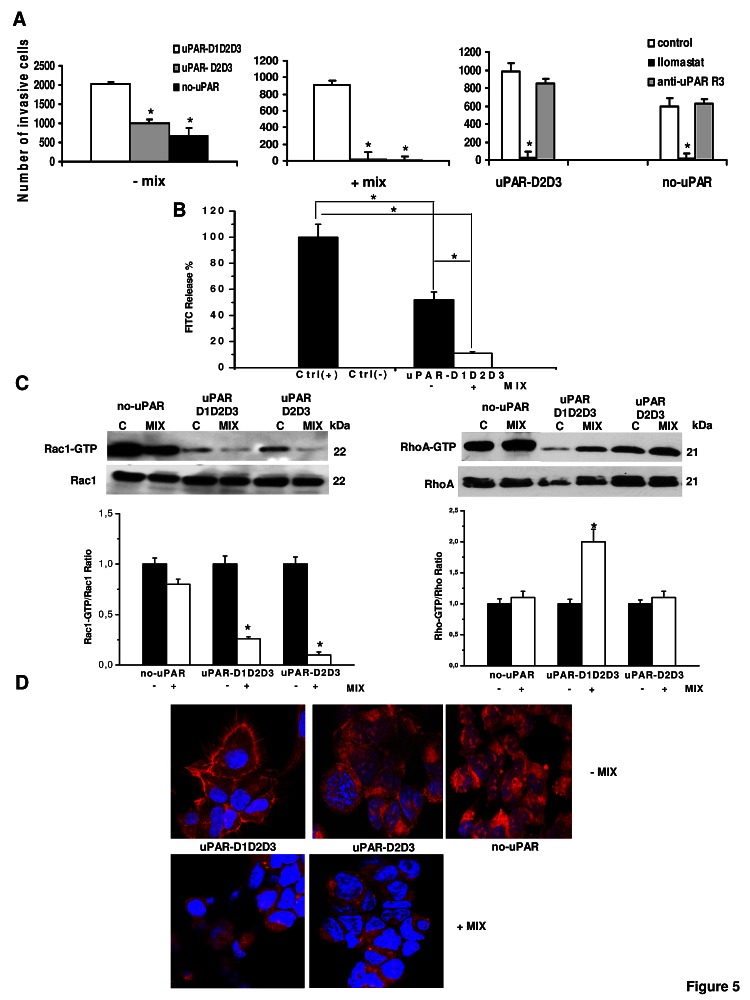
Mesenchymal and amoeboid features in HEK-293 cells transfected with intact uPAR (D1D2D3) and truncated uPAR (D2D3) Panel A: invasion of porous filters coated with a 3D Matrigel layer, showing the differential total number of invading cells/filter under mesenchymal and amoeboid conditions, as reported in the text. +/-Mix: presence or absence of the protease inhibitors cocktail. Ilomastat: invasion in the presence of the MMP inhibitor. uPARD1D2D3: HEK-293 cells transfected with intact uPAR; uPARD2D3: HEK-293 cells transfected with truncated uPAR; no uPAR: control cells transfected with an empty vector. Results are the mean of three different experiments performed in triplicate on each cell line ± SD. *: p<0.05 with respect to each control (first column of each histogram). Panel B: % collagenolytic activity of the uPARD1D2D3 transfectant under mesenchymal and amoeboid conditions, as described in the legend to figure 2. Ctrl-: collagenolytic activity in the absence of cells and of added exogenous collagen; Ctrl+: collagenolytic activity in the absence of cells but in the presence of exogenous collagen. Results are the mean of two different experiments performed in triplicate in each condition line ± SD. * : p<0.05. Panel C: Western blotting of total and GTP-loaded forms of small Rho-GTPases RhoA and Rac1 under mesenchymal and amoeboid conditions for each HEK-293 transfectant. Three experiments were performed for each transfectant in each condition. Histograms report band densitometry, assuming 1 as the reference value of controls in each condition. * shows statistical significance at p<0.05. Panel D: Morphological features of the mesenchymal (elongated) to amoeboid (roundish) transition (MAT) of each HEK-293 transfectant. Red: phalloidin staining of the actin cytoskeleton. Blue: nuclear staining with DAPI. Magnification: 200 X. Results shown are representative of two different preparations of each cell line under mesenchymal and amoeboid conditions. No-uPAR cells did not adhere under amoeboid conditions.

Native uPAR is organized in three differently folded homologous domains of about 90 amino acids each (D1, D2 and D3 from the N-terminus), stabilized by intra-molecular disulphide bonds [23]. The X-ray structure shows that uPAR binds uPA by directly interacting with D1, in a pocket built by all three domains [24]. These structural features suggest the necessity for cooperation of all three uPAR domains for high-affinity binding of uPA. uPAR may be anchored to the cell surface either in its native form (D1D2D3) or in a truncated form (D2D3), as a result of a cleavage of the D1-D2 linker region [25]. The uPAR-negative human embrional kidney-293 (HEK-293) cells transfected with cDNA of intact uPAR (uPAR-D1D2D3), with cDNAs corresponding to the truncated form of uPAR (uPAR-D2D3) and with pcDNA3 empty vector (no-uPAR) [26] were used to evaluate whether the native D1D2D3 or the cleaved D2D3 form of uPAR was involved in uPAR-dependent amoeboid movement style. The three differentially transfected cell lines were capable of invading Matrigel-coated filters, while only the uPAR-D1D2D3 cell line showed invasion capabilities upon exposure to a protease inhibitor-rich environment (figure 5A). This indicates that the presence of full length uPAR is strictly required in order to shift from the mesenchymal to amoeboid movement style. Invasion in the absence of protease inhibitors also shows that Hek-293 cells transfected with an empty vector (no-uPAR) and uPAR-D2D3 cells show similar invasion, although to a reduced level than uPAR-D1D2D3 (figure 5A on the left). Complete abolishment of invasion obtained in no-uPAR and in uPAR-D2D3 by Ilomastat treatment shows that Matrigel invasion of these cells is totally accounted for by MMPs, since it was inhibited by the MMPs inhibitor but not by the anti-uPAR R3 antibody (right panel of figure 5A). Also in these cells collagenolysis was inhibited by the mix of protease inhibitors (figure 5B). Experiments aimed to evaluate small GTPases Rac1 and RhoA activation showed a decrease of activated Rac1 in the presence of protease inhibitors in all the transfectants, while a significant increase of RhoA activation was observed only in uPAR-D1D2D3 cells (figure 5C). Morphological study of actin assembly of the three cell lines plated on VN-enriched Matrigel-coated surfaces showed the presence of actin protrusions and an overall spindle-like morphology only in full-length uPAR-expressing cells, while an overall feature of round cells was similarly exhibited by no-uPAR and uPAR-D3D3-expressing cells, which did not show actin protrusions (figure 5D). In a protease inhibitor-rich milieu no-uPAR cells were unable to adhere, while the totality of uPAR-D1D2D3 and uPAR-D2D3 cells assumed a round shape, lost actin filament protrusions and exhibited a rim of perinuclear cortical actin. Taken together, these data show that uPAR supports mesenchymal and amoeboid movement style only when expressed in its full-length native form.

### Integrins bridge uPAR and actin cytoskeleton

**Figure 6:**
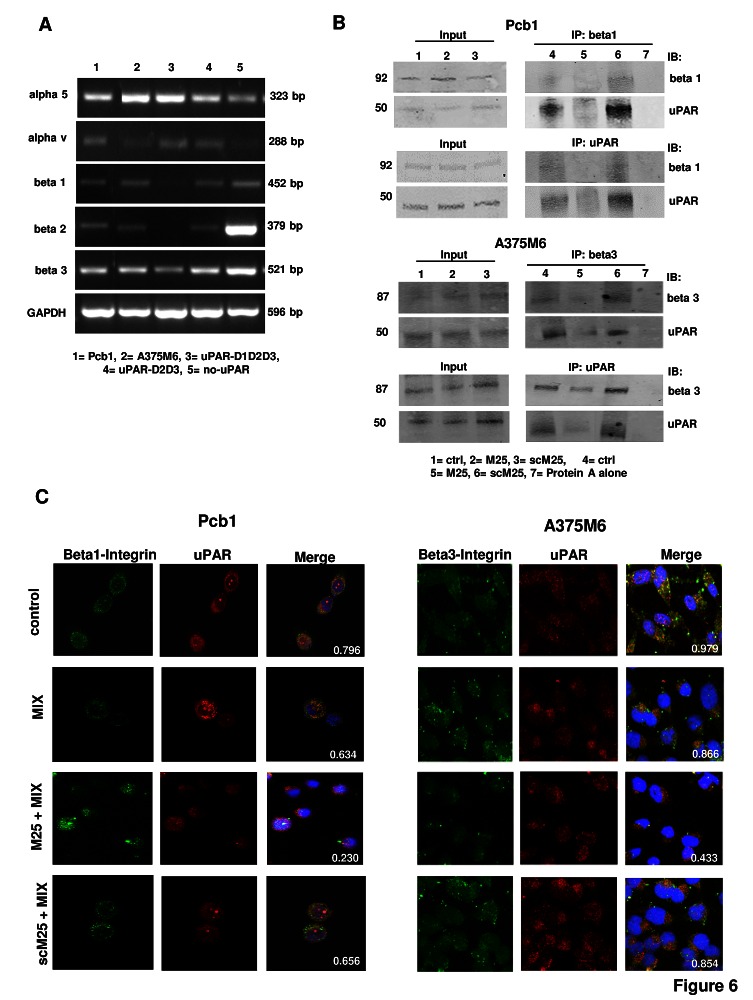
Integrin pattern and integrin-uPAR interaction Panel A: semiquantitative RT-PCR of the shown integrin α and β chains in the cell lines used in this study. GAPDH was used as a reference control. Products size, expressed in bp, are reported on the right. Panel B: immunoprecipitation of uPAR and β1-integrins in Pcb1 prostate carcinoma cells and of uPAR and β3-integrins in A375M6 melanoma cells. Input: Western blotting of aliquots of cell lysates before immunoprecipitation, used as a reference loading control. IP beta 1: immunoprecipitate obtained with anti-beta 1 antibodies; IB beta 1: immunoblotting with anti-beta 1 antibodies; IP beta 3: immunoprecipitate obtained with anti-beta 3 antibodies; IB beta 3: immunoblotting with anti-beta 3 antibodies; IB uPAR: immunoblotting with anti-uPAR antibody; IP uPAR: immunoprecipitate obtained with anti-uPAR antibodies. Molecular weights, expressed in kDa, are reported on the left. Panel C: confocal microscopy for uPAR (red fluorescence)-β1-integrins (green fluorescence) co-localization in Pcb1 prostate carcinoma cells and for uPAR (red fluorescence)- β3-integrins (green fluorescence) co-localization in A375M6 melanoma cells under mesenchymal (control) and amoeboid (+MIX) conditions, in the absence and in the presence of M25 peptide and of scramble M25 peptide (sM25). Nuclear staining: DAPI (blue). The co-localization score is reported within each picture. Refer also to table 1 for a complete view of co-localization scores in all the examined cell lines. Magnification: 40 X. The shown pictures are representative of 50 different pictures for each experimental condition that were studied by Image J analysis, as reported in the legend to table 1.

**Figure 7:**
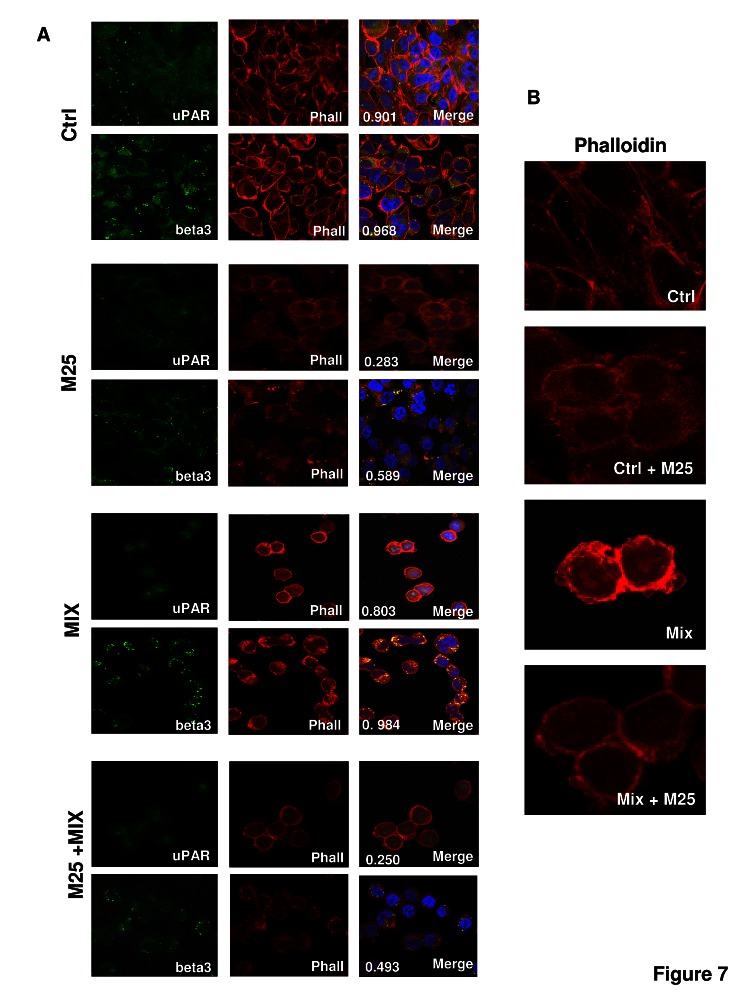
uPAR-actin co-localization Panel A: confocal microscopy for uPAR (green fluorescence) and falloidin (red fluorescence), in A375-M6 melanoma cells under mesenchymal (control) and amoeboid (+MIX) conditions, in the absence of M25 peptide, and in the presence of M25 peptide or of its scramble counterpart (sM25). The co-localization score is reported within each figure, as well as in table 1. Nuclear staining: DAPI (blue). Magnification: 40 X. The shown pictures are representative of 50 different pictures for each experimental condition that were studied by Image J analysis, as reported in the legend to table 1. Panel B: Magnification (200 X) of selected sections of panel A to show actin cytoskeleton (revealed by red falloidin staining) derangement as an effect of M25 peptide treatment.

Already established uPAR-mediated pathways include uPAR association with integrins, the key molecules that promote rearrangement of the actin cytoskeleton and cell movement.^10^ PCb1 cells, M6 melanoma cells and the three HEK-293 clones show an integrin pattern in line with previous observations (figure 6A) [26-30]. Confocal immuno-fluorescence analysis of integrin β1 and uPAR in PCb1 and of integrin β3 and uPAR in A375M6 cells is shown in figure 6C. These data show that uPAR-integrins interactions persist under both mesenchymal and amoeboid conditions. Treatment of cells with 50 μM peptide M25 for 2h at 37°C uncoupled uPAR from integrins while sM25 did not, in the absence and in the presence of the inhibitor cocktail (figure 6C). Immunoprecipitation experiments with lysates of Pcb1 and A375M6 cells demonstrated the activity of M25 peptide in uncoupling uPAR-integrin β1 or β3 interaction (figure 6B). The statistical analysis of uPAR-integrin co-localization for the cell lines considered in this study is reported in table 1. Parallel experiments aimed to evaluate uPAR-actin and integrins-actin colocalization showed that both uPAR and integrins co-localize with actin also in amoeboid conditions, where the thick ring of cortical actin/uPAR and cortical actin/integrin overlap (figure 7A). The use of peptide M25 uncoupled all the observed co-localizations (uPAR, integrins, actin) and produced structural alteration of actin which lost the stress fibres organization and acquired a granular feature under both mesenchymal and amoeboid conditions (figure 7B). The statistical analysis of uPAR-actin and integrin-actin co-localization for all the cell lines is reported in table 1.

**Table 1:** 

Antibody	Cells line & treatment	Colocalization coefficient (M2)
Anti-uPAR+Phalloidin	M6 ctrl	0,901
Anti-uPAR+Phalloidin	M6 + M25	0,283
Anti-uPAR+Phalloidin	M6 + mix	0,803
Anti-uPAR+Phalloidin	M6 + M25 + mix	0,250
Anti-uPAR+Phalloidin	Pcb1 ctrl	0,853
Anti-uPAR+Phalloidin	Pcb1 + M25	0,265
Anti-uPAR+Phalloidin	Pcb1 + mix	0,745
Anti-uPAR+Phalloidin	Pcb1 + M25 + mix	0,293

Anti-uPAR+Anti-Integrin β3	M6 ctrl	0,798
Anti-uPAR+Anti-Integrin β3	M6 + mix	0,713
Anti-uPAR+Anti-Integrin β1	Pcb1 ctrl	0,848
Anti-uPAR+Anti-Integrin β1	Pcb1 + mix	0,732

Anti-uPAR+Anti-Integrin β3	M6 ctrl	0,979
Anti-uPAR+Anti-Integrin β3	M6 + M25	0,542
Anti-uPAR+Anti-Integrin β3	M6 + mix	0,866
Anti-uPAR+Anti-Integrin β3	M6 + M25 + mix	0,433
Anti-uPAR+Anti-Integrin β1	Pcb1 ctrl	0,796
Anti-uPAR+Anti-Integrin β1	Pcb1 + M25	0,152
Anti-uPAR+Anti-Integrin β1	Pcb1 + mix	0,634
Anti-uPAR+Anti-Integrin β1	Pcb1 + M25 + mix	0,230

Anti-uPAR+Phalloidin	HEK uPAR 293	0,771
Anti-uPAR+Phalloidin	HEK uPAR 293 + mix	0,893
		
Anti-uPAR+Anti-Integrin β1	HEK uPAR 293	0,731
Anti-uPAR+Anti-Integrin β1	HEK uPAR 293 + mix	0,762

Anti-Integrin β3+Phalloidin	M6 ctrl	0,968
Anti-Integrin β3+Phalloidin	M6 + M25	0,589
Anti-Integrin β3+Phalloidin	M6 + mix	0,984
Anti-Integrin β3+Phalloidin	M6 + M25 + mix	0,493

M25 concentration = 50uM

### Activity of the M25 peptide on mesenchymal and amoeboid migration styles and related transductions

**Figure 8:**
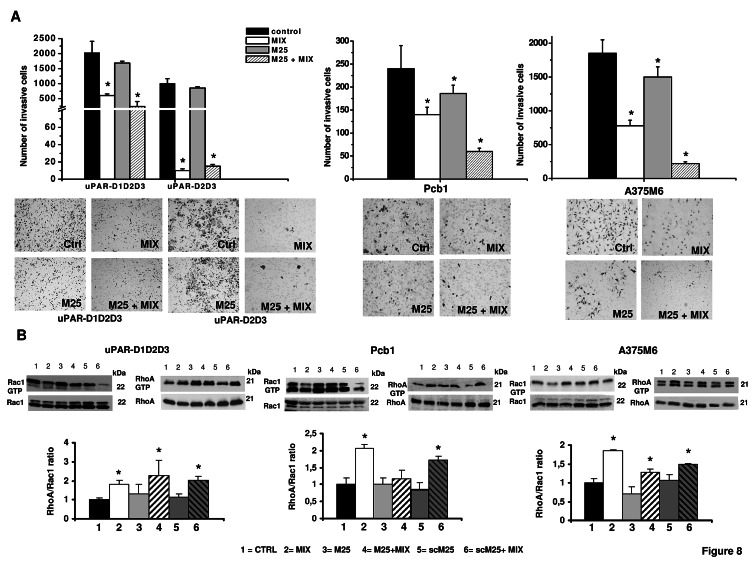
Phenotypic and biochemical effects of M25 treatment Panel A: invasion of porous filters coated with a 3D Matrigel layer, by HEK-293 transfectants, Pcb1 and M6 cells under amoeboid and mesenchymal conditions, in the absence and in the presence of M25 peptide. Results are the mean of three different experiments performed in triplicate on each cell line ± SD. *: p<0.05. The pictures show the typical appearance of filters in each experimental condition. Panel B: Western blotting of total and GTP-loaded forms of small Rho-GTPases Rac1 and RhoA under mesenchymal and amoeboid conditions ± M25 peptide for each prostate cancer and melanoma cell line. Three experiments were performed for each cell line in different conditions. Histograms report RhoA/Rac1 ratio obtained by band densitometry quantification and * shows statistical significance at p<0.05. Numbers on the right of each Western blotting refer to molecular weights expressed in kDa. For other symbols, refer to the legend of figure 1.

On the basis of these results we reasoned that the property of M25 peptide to uncouple uPAR from integrins, and hence from actin under both mesenchymal and amoeboid conditions, could produce functional effects similar to those obtained with uPAR aODN on mesenchymal and amoeboid migration styles. Therefore we studied Matrigel invasion and Rac-1/RhoA modulation under each relevant experimental condition. Similarly to genetic uPAR knockdown, results of Matrigel invasion obtained on PCb1, A375 M6 and Hek-293 cells clearly show that M25 peptide exerts a very intense inhibition of the amoeboid movement of cells treated with the inhibitor cocktail and partially inhibits the movement of control cells that move with a pre-specified mesenchymal migration style (figure 8A). Results obtained on small Rho-GTPases show that the shift from mesenchymal to amoeboid movement increases the RhoA/Rac1 ratio, even in the presence of M25 peptide (figure 8B), but such a Rho over-activation does not support amoeboid movement owing to the M25-dependent weakening of actin cytoskeleton-integrins-uPAR functional axis. Overall, the phenotypic effects obtained with the M25 peptide are similar to those obtained with uPAR-aODNs, allowing us to conclude that uPAR regulates plasticity of cell movement in both the mesenchymal and amoeboid migration styles by an integrin-mediated coupling of the cell membrane to the actin cytoskeleton.

## DISCUSSION

A ten years analysis of the prospective multicentre Chemo-N0 (node negative) trial has identified the uPA/uPAR system as the only level-of-evidence-1 cancer biomarker system for prognosis and/or prediction in node-negative breast cancer [31]. uPAR contributes to multiple features of the malignant character, including high metastatic potential [32], angiogenesis [33], epithelial-to-mesenchymal-transition (EMT) [34] and cancer cell stemness [35]. Our data show that uPAR has a role in sustaining both mesenchymal and amoeboid migration style of cancer cells by controlling the state of the actin cytoskeleton assembly via its integrin-mediated interaction with cytoskeleton.

Far-off and recent studies have underlined the key role of multiple proteases and of their receptors in mesenchymal migration style of cancer cell during the invasion process so that the property of cancer cells to proteolytically degrade anatomical barriers has attained the role of a marker of cancer cell malignancy [36,37]. Amoeboid motility has been originally described in the amoeba *Dictyostelium discoideum*, but it is also exploited by eukaryotic cells under particular environmental cues, such as the need to rapidly slide through lose interstitial tissues with gaps that accommodate the cell body using flexible and weak adhesion sites, producing a movement that depends on dynamic cytoplasmic protrusions due to intracellular compartmentalization of cytoplasm between an intact cortical actin cytoskeleton and the locally expanded cell membrane (blebs) [38]. Pioneer studies enlightened the possibility of cancer cells to move within tissues according to amoeboid features, leading to the so-called “cortical expansion model for amoeboid chemotaxis” of malignant cells [39]. Cancer cell amoeboid motility has now become an issue, accounting for rapid movement of invading cells within the primary and the metastatic site.

Herein we have shown that malignant cells derived from human melanoma and prostate cancer, as well as human embrional kidney 293 cells (HEK-293) transfected with the full-length form (D1D2D3) of the human uPAR, exhibit a uPAR-dependent mesenchymal invasion across a 3D Matrigel substrate, that is proportional to uPAR expression and that may be strongly reduced by preventing uPAR interaction with its ligand uPA, the observed residual mesenchymal Matrigel invasion being surely ascribable to the activity of multiple proteases. Our results indicate that the uPAR-driven proteolytic activity accounts for more that 50% of single migrating cells in all the studied cancer cell lines. The residual cell invasion has to be related to many other proteases such as members of the MMP and cathepsin families. Due to the high number of involved molecules it was not possible to use blocking antibodies, while the use of specific protease inhibitors could induce the shift to an amoeboid movement style. The same cells shifted toward an amoeboid migration style (MAT) upon exposure to a protease inhibitor cocktail. Our observation that not the whole cancer cell populations undergo MAT and that the percentage of shifting cells varies among the different cell lines independently of their origin (primary tumor or metastasis) indicates that this feature is independent of cancer progression. Also small Rho-GTPases activation and cell morphology were consistent with MAT. Although under mesenchymal conditions not all the studied cell lines showed an unambiguously elongated cell shape, the acquisition of amoeboid features in the presence of the inhibitor cocktail, such as the attainment of a round shape and reorganization of actin according to a strictly sub-cortical location were evident.

The results obtained with uPAR-aODN on mesenchymal and amoeboid movement clearly show uPAR requirement in both styles of cancer cell migration. Moreover, the data obtained with HEK-293 cells transfected with the truncated or with the native form of uPAR show that only the full-length form supports amoeboid movement. Many proteases cleave the receptor (cleaved uPAR, c-uPAR) [40]. c-uPAR, devoid of D1, is unable to bind extracellular uPAR ligands, uPA and VN, as well as to interact with integrins, indicating that uPAR cleavage is a mechanism that negatively regulates uPAR activities associated with a migratory and invasive phenotype: in fact the full-length uPAR and uPA are up-regulated and are negative prognostic factors in several tumors [31]. In order to elucidate the mechanism whereby native uPAR regulates amoeboid movement, we decided to explore the in-ward uPAR activities by studying its property to regulate the actin cytoskeleton [10,11,26,41]. Interaction of uPAR with integrins is the foundation stone of the cell migration signalosome [10]. In our cancer cells we have confirmed uPAR interaction with β1 and β3 integrins by confocal microscopy and immunoprecipitation, showing that such interactions are present under both mesenchymal and amoeboid conditions. Integrins, which lack actin-binding domains, are indirectly connected to the actin cytoskeleton through different protein complexes that contain over a hundred different types of proteins, talin, α-actinin, filamin, tensin, parvin and myosin-X being the most studied, that transmit the mechanical force from the cell membrane to the actin cytoskeleton for cytoplasm contraction during cell movement [15]. We have shown herein that uPAR co-localizes with actin cytoskeleton in all the examined conditions, including the amoeboid one, where the thick ring of cortical actin and uPAR overlap. In the β-propeller model of α-chain folding, the sequence of the M25 peptide spans an exposed loop on the uPAR-binding surface of α-chain, thus impairing integrin-uPAR interaction [42]. M25 peptide not only uncoupled uPAR from integrins in all the cells and experimental conditions studied in this work, but it also produced a structural alteration of actin which lost the stress fibres and cortical organization under the mesenchymal and amoeboid conditions, and acquired a granular feature. It is therefore evident that M25 peptide uncouples uPAR from integrins, and hence from actin, thereby producing functional effects similar to those obtained with uPAR aODN on mesenchymal and amoeboid migration styles. The final effects of both treatments, that lead to an alteration of actin assembly, which is no longer efficiently bound to integrins modified by the loss of interaction with uPAR, result into a weakening of actin-membrane coupling and in the loss of the tensile force that determines translocations of the cell body [15]. Actin disorganization impaired both mesenchymal and amoeboid migration of cancer cells. Also the activity of small Rho-GTPases was deranged by the use of M25 peptide as well as by uPAR-aODN, thus partially inhibiting mesenchymal migration and totally inhibiting the amoeboid one. As the M25 peptide is specific for inhibition of uPAR-integrin α-chain interaction, its limited effects of on mesenchymal migration may be accounted for by the failure of the peptide to inhibit integrin-MMPs interactions that also regulate mesenchymal motility [43,44].

Available data indicate that a successful strategy to combat invasion and metastatic diffusion of aggressive cancer cells is the identification of molecular targets that control the ability of cancer cells to adapt to the environment by regulating plasticity of cancer cells. Taken together, the results obtained in the present study show that uPAR, a recognized marker of cancer cell progression, is a molecular mediator of plasticity in cancer cell motility by regulating contractile forces in tumor cell migration through the functional axis uPAR-integrins-actin. Together with the many so far reported pro-tumoral activities of uPAR, our observations make the uPA/uPAR system an attractive target for the treatment of cancer that has not yet been extensively explored in the clinic [45].

## MATERIALS AND METHODS

### Cell lines and culture conditions

Prostate cancer cell lines LNCaP and PC3 were purchased from ATCC (Manassas, VA, USA). LNCaP are androgen-sensitive human prostate adenocarcinoma cells, while PC3 cells, derived from a bone metastasis of a grade IV prostatic adenocarcinoma, are reported to be androgen receptor (AR)-negative. Pcb1 cell line, established from a bone metastasis of PC3, were provided by Professor Adriano Angelucci (L'Aquila, Italy). All prostate cancer cell lines were grown in RPMI-1640 culture medium (Euroclone, Milano, Italy) containing 2 mM glutamine, 100 UI/ml penicillin, 100 μg/ml streptomycin and supplemented with 10% fetal bovine serum (FBS; Euroclone). Melanoma cell lines MeWo and A375 were purchased from ATCC. A375 M6 cells, derived from a metastasis of A375, were provided by Professor Lido Calorini (Firenze, Italy). Melanoma cells were maintained in DMEM culture medium (Euroclone, Milano, Italy) containing 2 mM glutamine, 100 UI/ml penicillin, 100 μg/ml streptomycin and supplemented with 10% FBS (Euroclone). uPAR-negative human embrional kidney 293 (HEK-293) cells transfected with cDNA of intact uPAR (uPAR-D1D2D3), with cDNAs corresponding to the truncated form of uPAR (uPAR-D2D3) and with pcDNA3 empty vector (no-uPAR), were kindly provided by Professor Pia Ragno (Napoli, Italy). HEK-293 transfected cells were grown in DMEM culture medium (Euroclone, Milano, Italy) containing 2 mM glutamine, 100 UI/ml penicillin, 100 μg/ml streptomycin and supplemented with 10% fetal bovine serum (FBS; Euroclone).

### Quantitative Real-Time PCR analysis

Total RNA was prepared using Nucleospin RNA II (Macherey-Nagel), agarose gel checked for integrity, and reverse transcribed with GoScript system (Promega) using random primers according to manufacturer's instructions. uPAR expression in all cancer cell lines and after anti-uPAR aODN treatment was determined by a quantitative Real-Time (RT)–PCR with an Applied Biosystem 7500 Fast Real Time PCR System (Applied Biosystems, Milano, Italy) and determined by the comparative C_t_ method using 18S ribosomal RNA as the normalization gene. Amplification was performed with the default PCR setting: 40 cycles of 95°C for 15 seconds and of 60°C for 60 seconds using SYBR Green–based detection (GoTaq qPCR Master Mix; Promega). Primers (IDT, Tema Ricerca, Italy) used for RT-PCR were as follows: -18S-rRNA: sense, 5'-CCAGTAAGTGCGGGTCATAAG-3' antisense, 5'-GCCTCACATAACCATCCAATC-3'; -uPAR: sense, 5'- GCCCAATCCTGGAGCTTGA-3; antisense, 5'- TCCCCTTGCAGCTGTAACACT-3'.

### Semiquantitative reverse transcription–polymerase chain reaction (PCR) analysis

Total RNA preparation and reverse transcription were performed as previously reported [20]. The levels of messenger RNA for the integrin chains were determined by an internal-based semiquantitative RT-PCR, using procedures and primers previously described [13].

### 3D-invasion assay with Boyden chambers

Invasion was studied in Boyden chambers in which the upper and lower wells were separated by 8μm–pore size polycarbonate filters coated with Matrigel (BD Biosciences). In order to increase uPAR dependency of the invasion process, the Matrigel was enriched with human VN (Sigma-Aldrich, cat. V8379), to a final concentration of 300 μg/ml. To obtain a “thick” layer, we added 100 μl of Matrigel/cm^2^ porous membrane. According to the manufacturer's instruction such an amount results into a 500 μm thick Matrigel layer, corresponding to about 25 folds the average diameter of circulating prostate and melanoma cells [46,47]. Cancer cells (25 x 10^3^) were placed in the upper well of the Boyden chamber, and invasion was performed at 37 °C in 5% CO2 for 12 hours, a time consistent with the average speeds of cell migration under amoeboid and mesenchymal conditions in 500 μm thick gels [2]. To evaluate the uPAR-dependent invasion, some experiments were performed in the presence of anti-uPAR R3 antibody which blocks uPA/uPAR interaction (American Diagnostica) or irrelevant IgG (all used at 1.5 μg/ml) or after anti-uPAR aODN treatment. Invasion quantified as previously described [21,22].

### Induction of the amoeboid phenotype, cell viability assay and collagen degradation assay

Protease-indipendent invasion was evaluated by 3D-Boyden chamber assays with Matrigel coating in the presence of a protease inhibitor cocktail, consisting of Ilomastat, leupeptin, pepstatin A, E-64 and aprotinin, a mixture of protease inhibitors with a broad specificity for serine, cysteine, aspartic and amino-peptidase, and MMPs [7]. Protease inhibitor cocktail was added to un-polymerized Matrigel solution on the upper surface of the porous filter. To induce the amoeboid phenotype, cells were treated for 2 hours with the protease inhibitor cocktail at the same concentrations used in the invasion assay. The cocktail did not affect cell viability, as shown by WST-1 assay (Roche), performed as previously described [20].

The collagen degradation assay was performed using a Matrigel layer containing 3% FITC-labelled collagen monomers, as reported previously [7].

### Cell migration in 3D-collagen matrices

To visualize the efficacy of the protease inhibitor cocktail to induce an amoeboid migration style we used reconstruction by time-lapse video microscopy and confocal microscopy, using HT1080 cells as a model for a clear visualization of fiber breakdown in the process of proteolytic migration and shift to the amoeboid style, as previously reported [7]. (SM 1 and 2).

### RhoA and Rac1 activity assay

Cells from different experimental conditions (control, protease inhibitor cocktail, anti-uPAR aODN and anti-uPAR aODN + protease inhibitor cocktail) were lysed in radioimmunoprecipitation assay buffer and determination of RhoA GTP or Rac1 GTP was performed as previously reported [7].

### Western Blotting

The composition of the lysis buffer was as previously described [42]. The same lysis buffer was used to solubilise beads-adsorbed active forms. Both beads and cell proteins were subjected to Western blotting as previously reported [22]. The membrane was probed with primary antibody to uPAR, RhoA and Rac1 (Becton Dickinson), overnight at 4° C. Immune complexes were detected as previously reported [22].

### Immunofluorescence analysis

Immunofluorescence was performed as previously described [13,22]. Cells were grown on coverslips in their culture conditions. Once at confluence, cells were treated with protease inhibitor cocktail and Ilomastat. Two hours later TRITC-labelled phalloidin (P1951, Sigma) was applied to the cells to visualize cell morphology and the arrangement of actin cytoskeleton. Nuclei were stained with the fluorescent Hoechst 33342 dye (DAPI) (10μg/ml) (Invitrogen) for 15 min at rt. A single composite image was obtained by superimposition of twenty optical sections for each sample observed. The collected images were analysed by ImageJ software to evaluate co-localization.

### uPAR down-regulation with antisense oligodeoxinucleotide (u-PAR aODN)

uPAR gene expression was inhibited with an 18-mer phosphorothioate aODN (ISIS Pharmaceuticals, Carlsbad Research Center, CA, USA, product designation: ISIS 17916) as previously described [21].

### Treatment of cells with M25 peptide

Disruption of uPAR-integrin interaction was obtained with the M25 peptide, previously identified in a phage display library [42,48], able to uncouple uPAR interaction with integrin α-chain. The peptide was produced in collaboration with PRIMM srl, Milan, Italy. In the β-propeller model of α-chain folding, the sequence of this peptide (STYHHLSLGYMYTLN) spans an exposed loop on the ligand-binding surface of α-chain, thus impairing integrin-uPAR interaction. In cell culture both M25 and scramble-M25 (sM25) were used at 50 μM for 2h at 37°C.

### Statistical analysis

Results are expressed as means ± SD. Multiple comparisons were performed by the Student test. Statistical significances were accepted at p<0.05.

## SUPPLEMENTARY INFORMATIONS




