# Correction: The receptor for urokinaseplasminogen activator uPAR controls plasticity of cancer cell movement in mesenchymal and amoeboid migration style

**DOI:** 10.18632/oncotarget.28871

**Published:** 2026-05-20

**Authors:** Francesca Margheri, Cristina Luciani, Maria Letizia Taddei, Elisa Giannoni, Anna Laurenzana, Alessio Biagioni, Anastasia Chillà, Paola Chiarugi, Gabriella Fibbi, Mario Del Rosso

**Affiliations:** ^1^Department of Experimental and Clinical Biomedical Sciences, University of FlorenceIstituto Toscano Tumori; ^*^These authors contributed equally to the study

**This article has been corrected:** It was found that the first lane of PCR bands in Figure 6A, was spliced with the subsequent line. The authors explained that bands were removed from the original gel image to exclude a cell line that was ultimately not included in the published study. This splice has now been clearly marked.

Additionally, an overlap was noted between the MIX and scM25+MIX panels in the A375M6 melanoma cell immunofluorescent images in Figure 6C. The authors have provided the original data for the scM25+MIX panel, which was used to correct this figure.

The corrected Figure 6, produced using the original data, is shown below. The authors declare that these corrections do not change the results or conclusions of this paper.

Original article: Oncotarget. 2014; 5:1538–1553. 1538-1553. https://doi.org/10.18632/oncotarget.1754

**Figure 6:**
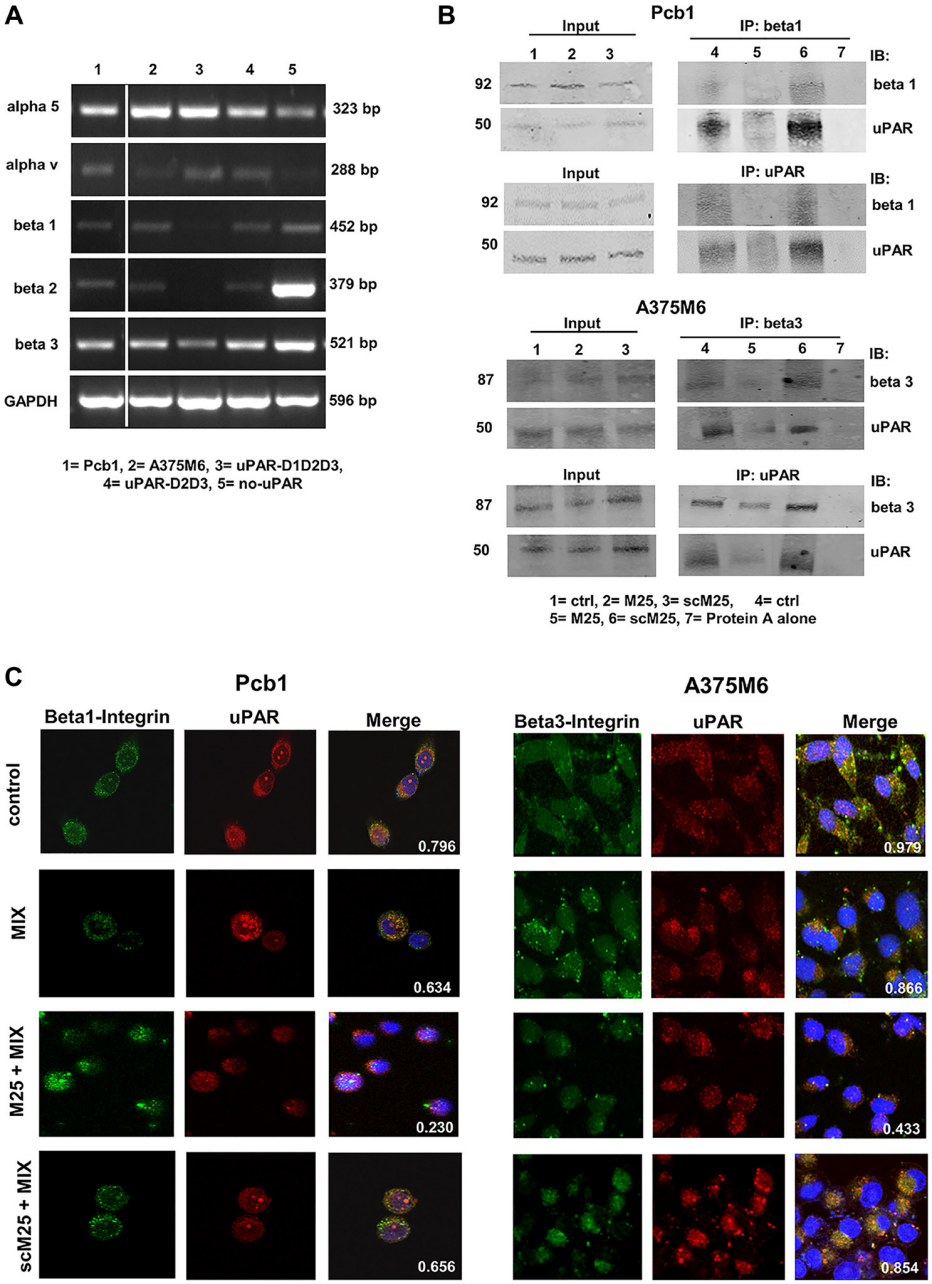
Integrin pattern and integrin-uPAR interaction. (**A**) Semiquantitative RT-PCR of the shown integrin α and β chains in the cell lines used in this study. GAPDH was used as a reference control. Products size, expressed in bp, are reported on the right. (**B**) Immunoprecipitation of uPAR and β1-integrins in Pcb1 prostate carcinoma cells and of uPAR and β3-integrins in A375M6 melanoma cells. Input: Western blotting of aliquots of cell lysates before immunoprecipitation, used as a reference loading control. IP beta 1: immunoprecipitate obtained with anti-beta 1 antibodies; IB beta 1: immunoblotting with anti-beta 1 antibodies; IP beta 3: immunoprecipitate obtained with anti-beta 3 antibodies; IB beta 3: immunoblotting with anti-beta 3 antibodies; IB uPAR: immunoblotting with anti-uPAR antibody; IP uPAR: immunoprecipitate obtained with anti-uPAR antibodies. Molecular weights, expressed in kDa, are reported on the left. (**C**) Confocal microscopy for uPAR (red fluorescence)-β1-integrins (green fluorescence) co-localization in Pcb1 prostate carcinoma cells and for uPAR (red fluorescence)- β3-integrins (green fluorescence) co-localization in A375M6 melanoma cells under mesenchymal (control) and amoeboid (+MIX) conditions, in the absence and in the presence of M25 peptide and of scramble M25 peptide (sM25). Nuclear staining: DAPI (blue). The co-localization score is reported within each picture. Refer also to Table 1 for a complete view of co-localization scores in all the examined cell lines. Magnification: 40 X. The shown pictures are representative of 50 different pictures for each experimental condition that were studied by ImageJ analysis, as reported in the legend to Table 1.

